# Supplementary material for: Insights into the inhibition of protospacer integration via direct interaction between Cas2 and AcrVA5
Source: Nat Commun. 2024 Apr 16;15:3256. doi: 10.1038/s41467-024-47713-7 (PMC11021501; doi:10.1038/s41467-024-47713-7)
Supplement: Supplementary file 1 — Supplementary Information [file 41467_2024_47713_MOESM1_ESM.pdf]

Supplementary Material for

Insights into the inhibition of protospacer integration via direct interaction between Cas2 and AcrVA5

Mingfang Bi[1], Wenjing Su[1], Jiafu Li[1] and Xiaobing Mo[1,2,*]

*: To whom correspondence should be addressed to Xiaobing Mo, Email: mox@jlu.edu.cn.

Table S1. The buried surface area in the complex of Cas2 and AcrVA5-peptide

**Surface area of Cas2**	11785.87 Å^2
**Surface area of peptide**	785.26 Å^2
**Buried surface area**	434.56 Å^2

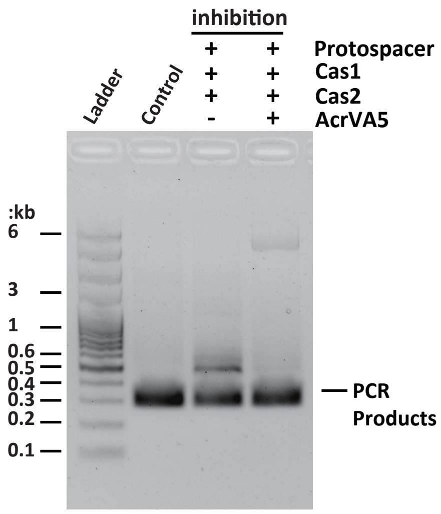

Supplementary Figure 1. ***In vivo*** **inhibition the protospacer integration**

This figure demonstrates the effects of *in vivo* inhibition of AcrVA5 on protospacer integration by Cas1-Cas2.

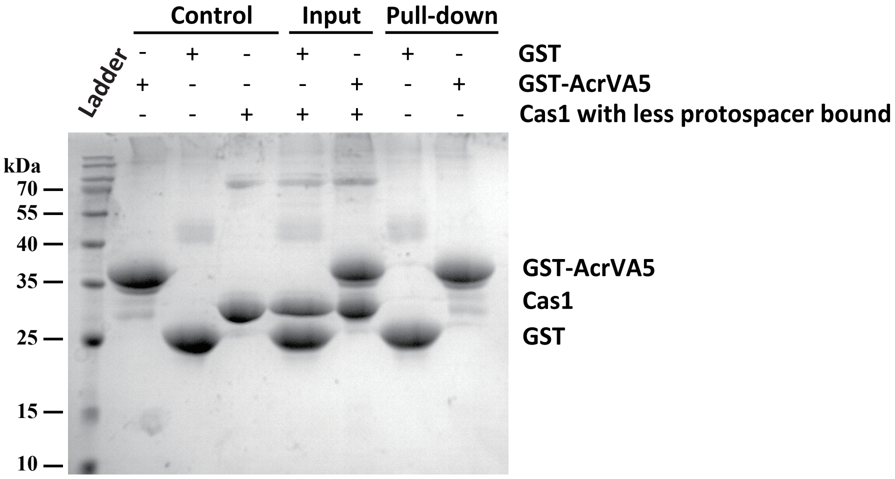

Supplementary Figure 2. ***In vitro*** **GST pull-down of Cas1 with AcrVA5**

This figure displays the results of an *in vitro* GST pull-down assay. The lanes represent distinct experimental groups: control, input, and pull-down. In this setup, the Cas1 protein is pulled down with GST-tagged AcrVA5. The data allows for the conclusion that no specific protein-protein interactions occur between Cas1 and AcrVA5.

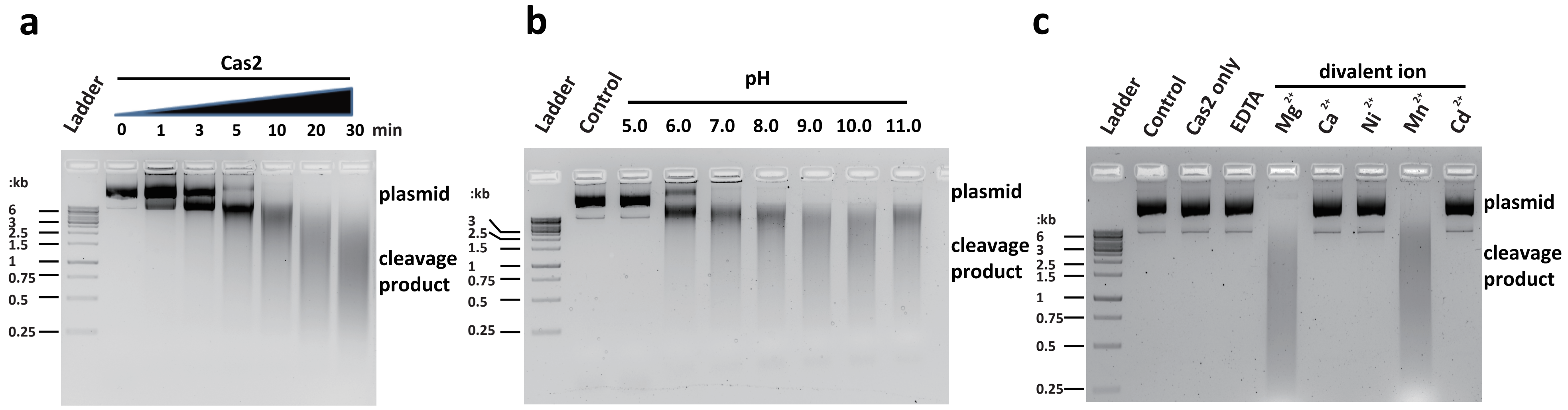

Supplementary Figure 3. The endonuclease activity of Cas2

(a) This part of the figure illustrates the progression over time of Cas2's nuclease activity.

(b) This section demonstrates that the dsDNA nuclease activity in Cas2 is pH-dependent, indicating that changes in pH can significantly affect this activity.

(c) This panel shows that the dsDNA nuclease activity in Cas2 is influenced by the presence of divalent ions. Specifically, Mg^{2+} and Mn^{2+} dramatically enhance the activity, while the addition of EDTA inhibits the dsDNAse activity, underscoring the importance of ion balance in enzyme function.

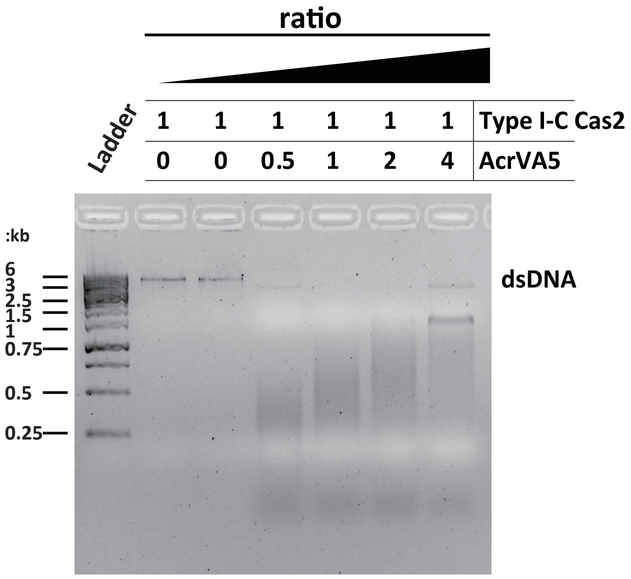

Supplementary Figure 4. Inhibition of *Mb*-Cas2's endonuclease activity by *Mb*-AcrVA5. This figure provides a graphical representation of the inhibitory effect of AcrVA5 on the endonuclease activity of Cas2. The result displays a decrease in Cas2's endonuclease activity with increasing amounts of AcrVA5, demonstrating a clear inhibitory effect. This figure effectively illustrates the role of AcrVA5 in modulating the enzymatic activity of Cas2.

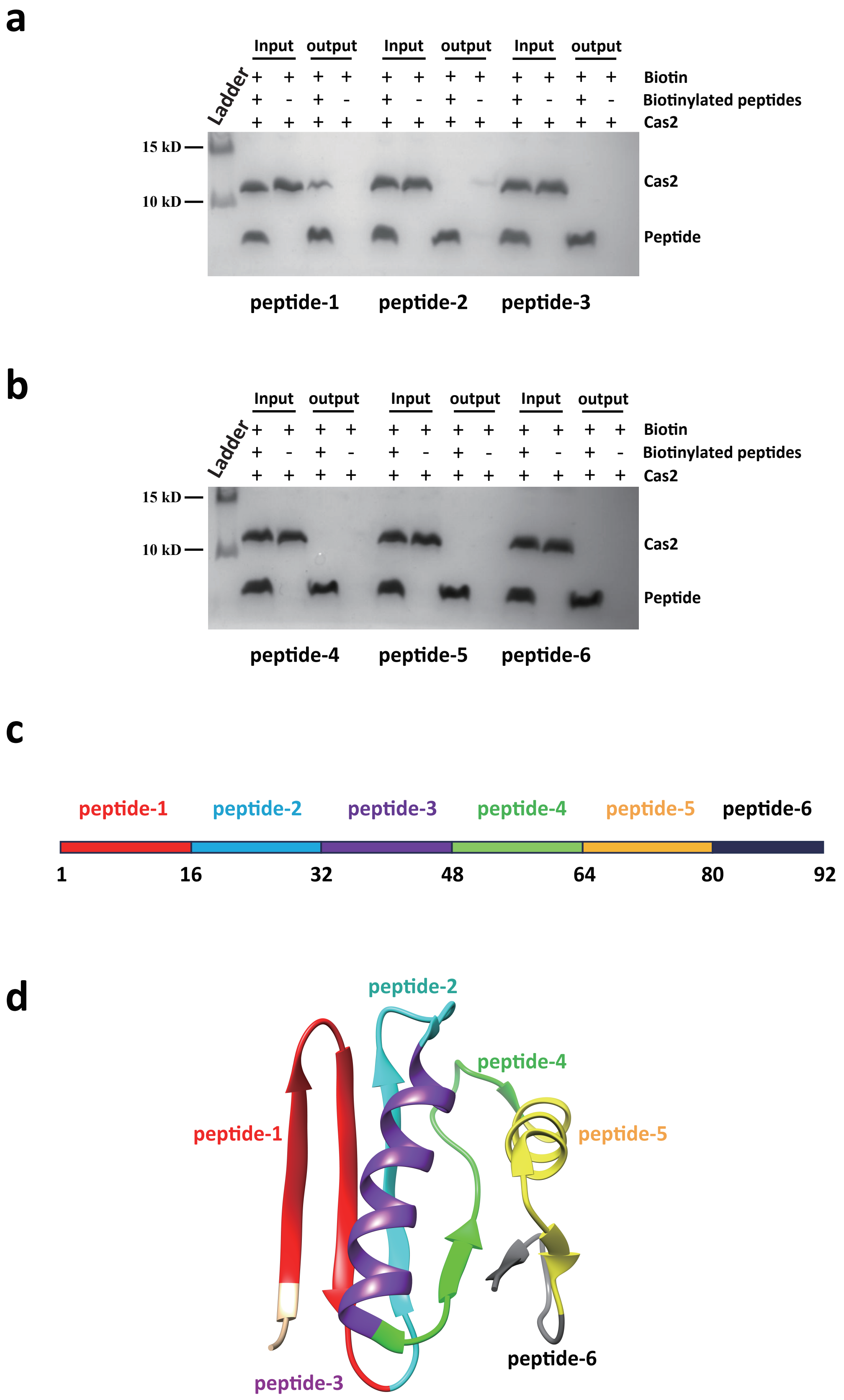
a
Input output Input output Input output
Ladder
Biotin
Biotinylated peptides
Cas2
15 kD
10 kD
Cas2
Peptide
peptide-1 peptide-2 peptide-3
b
Input output Input output Input output
Ladder
Biotin
Biotinylated peptides
Cas2
15 kD
10 kD
Cas2
Peptide
peptide-4 peptide-5 peptide-6
c
peptide-1 peptide-2 peptide-3 peptide-4 peptide-5 peptide-6
1 16 32 48 64 80 92
d
peptide-2
peptide-4
peptide-1
peptide-5
peptide-6
peptide-3

Supplementary Figure 5. Peptide-scanning affinity capture

(a, b) The interaction between Cas2 and AcrVA5-peptides were examined using an *in vitro* affinity capture assay. The biotinylated AcrVA5-peptides were bound to streptavidin beads and subsequently incubated with the Cas2 protein. Following extensive washing to remove non-specifically bound material, the remaining resolved protein was analyzed using SDS-PAGE. The proteins were then visualized using Coomassie brilliant blue staining. This approach effectively demonstrates potential interactions between Cas2 and AcrVA5-peptides. (c) Segmentation of the full-length AcrVA5 is presented. (d) The sequences of peptides in the structure of AcrVA5 are depicted.

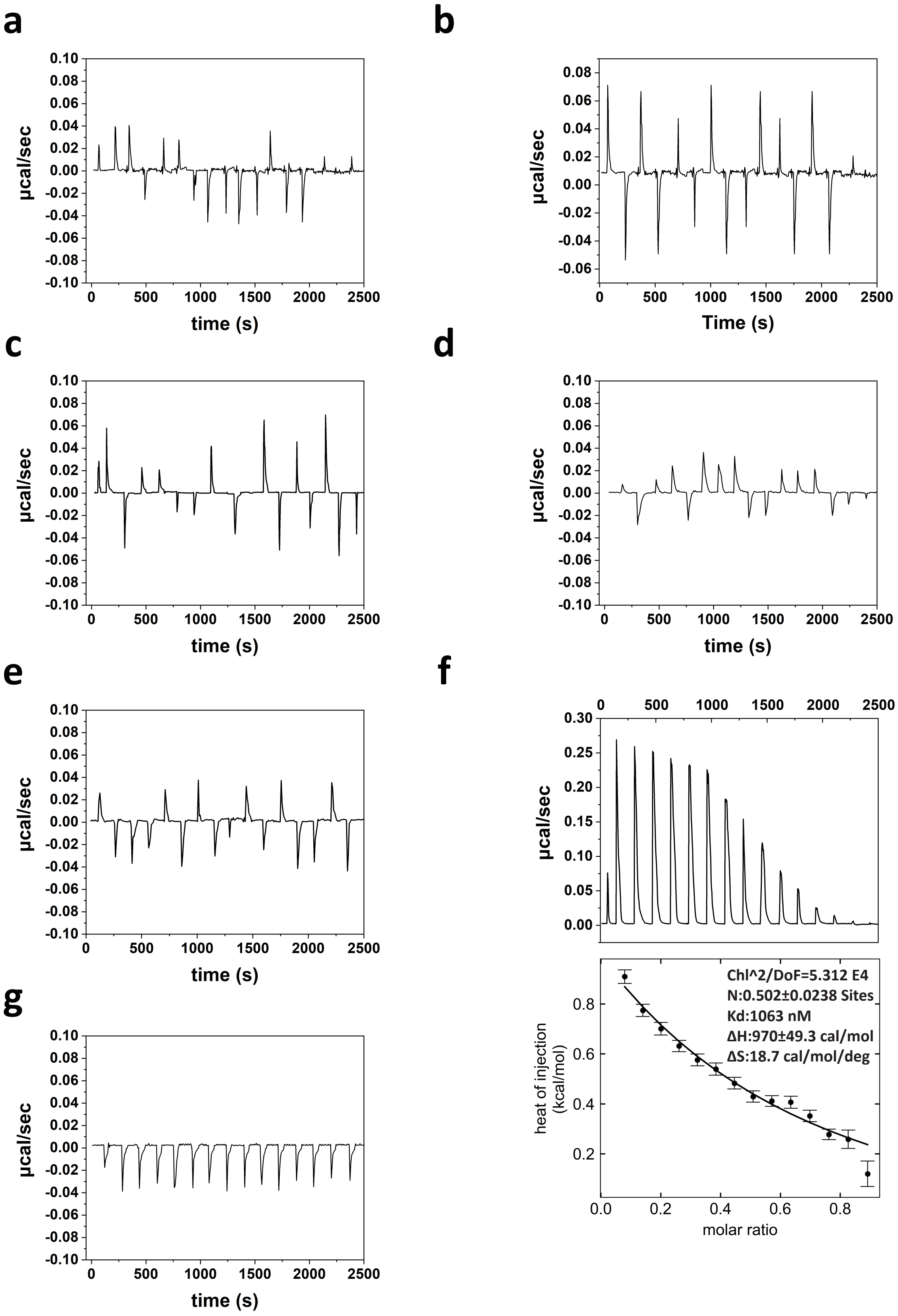
a
b
c
d
e
f
g
µcal/sec
time (s)
Time (s)
heat of injection (kcal/mol)
molar ratio
Chi^2/DoF=5.312 E4
N:0.502±0.0238 Sites
Kd:1063 nM
ΔH:970±49.3 cal/mol
ΔS:18.7 cal/mol/deg

Supplementary Figure 6. ITC of Cas2 to AcrVA5-peptide / AcrVA5-mutants

(a) ITC of Cas2 to AcrVA5-peptide-2. (b) ITC of Cas2 to AcrVA5-peptide-3. (c) ITC of Cas2 to AcrVA5-peptide-4. (d) ITC of Cas2 to AcrVA5-peptide-5. (e) ITC of Cas2 to AcrVA5-peptide-6. The weak or possibly nonexistent interaction between Cas2 and the AcrVA5-peptides is suggested by the acquired data. (f) The dissociation constant (Kd) signifying the interaction between AcrVA5-L5A and Cas2 is calculated to be ~1.3 μM. This value suggests a relatively weak interaction between these two proteins. (g) ITC of Cas2 to AcrVA5 with N-terminal M1-G7 deletion.

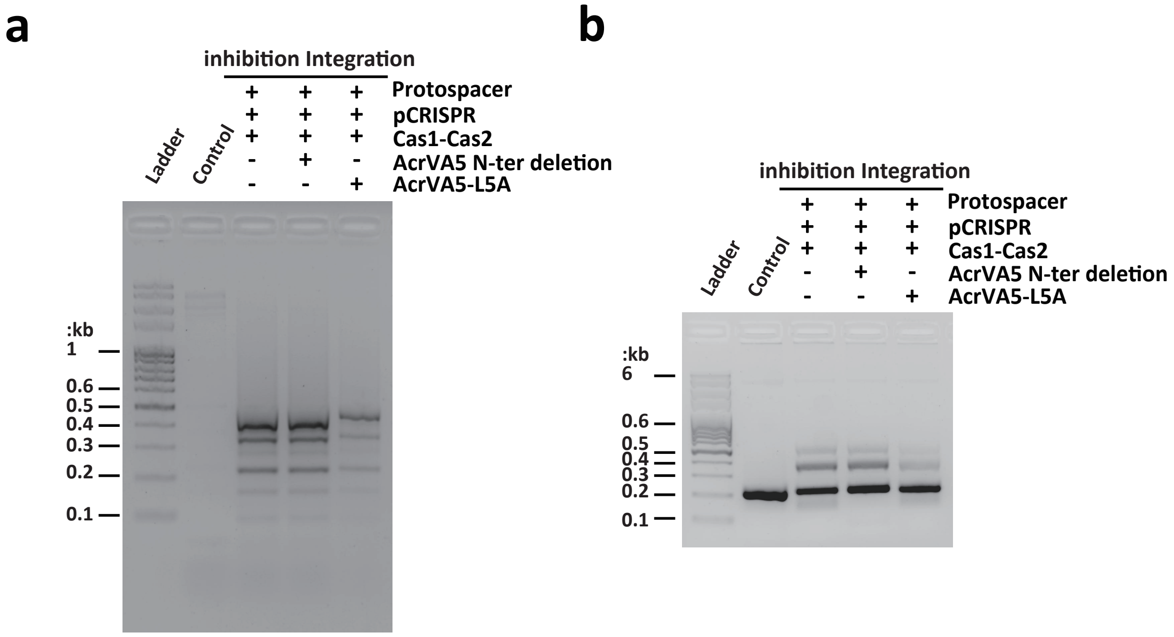

Supplementary Figure 7. ***In vitro*** **and** ***in vivo*** **integration assays**

(a) *In vitro* integration assays by AcrVA5-L5A or AcrVA5 N-ter deletion.

(b) *In vivo* integration assays by AcrVA5-L5A or AcrVA5 N-ter deletion. The resulting electrophoretic profiles illustrate the functional importance of the N-terminal region of AcrVA5 for Cas2 binding. The results underscore the varying degrees of protospacer integration in the presence of AcrVA5-mutants, hinting at their regulatory role in the integration process.

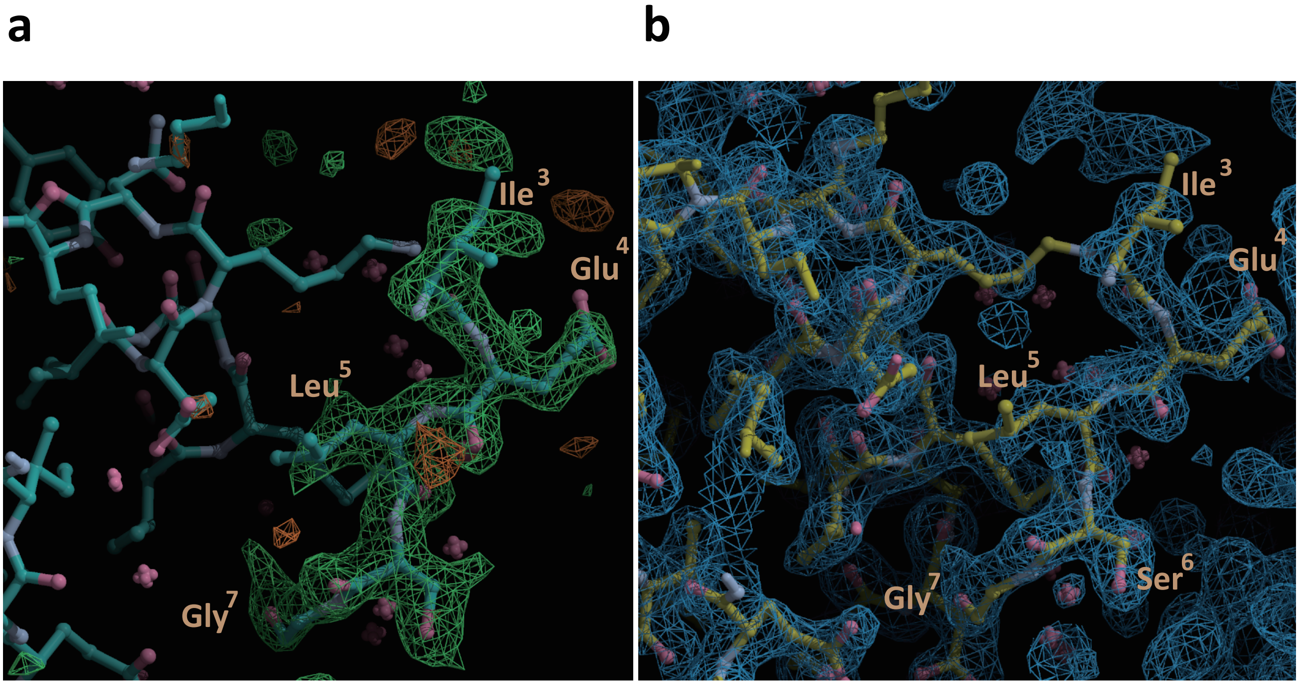

Supplementary Figure 8. The Omit map of the AcrVA5-peptide in the complex structure.

(a) This figure depicts the omit map of the AcrVA5-peptide within the complex structure. The electron density map, shown in mesh, reveals the precise positioning of the AcrVA5-peptide. The Acrva5-peptides are represented in stick models with specific color coding indicating different atoms. The figure clearly visualizes spatial arrangement of the AcrVA5-peptide within the complex, highlighting its crucial role in the structure.

(b) The 2mFo - DFc map is presented, featuring labels that indicate key amino acid residues.

a

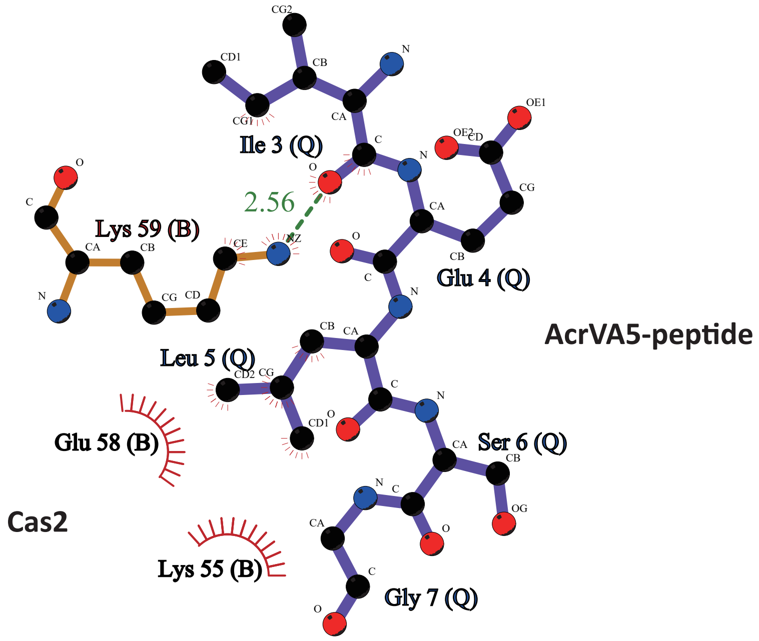

b

Hydrogen bonds:

Atom-1					Atom-2				
Atom	Name	No.	Chain		Atom	Name	No.	Chain	Distance
NZ	Lys	59	B	------	O	Ile	3	Q	2.56

Non-bonded contacts:

Atom-1					Atom-2				
Atom	Name	No.	Chain		Atom	Name	No.	Chain	Distance
CG	Lys	55	B	------	CB	Leu	5	Q	3.72
CG	Lys	55	B	------	CG	Leu	5	Q	3.88
CG	Lys	55	B	------	CD1	Leu	5	Q	3.44
CG	Glu	58	B	------	GG	Leu	5	Q	3.67
CG	Glu	58	B	------	CD2	Leu	5	Q	3.66
CE	Lys	59	B	------	O	Ile	3	Q	3.24
NZ	Lys	59	B	------	C	Ile	3	Q	3.61
NZ	Lys	59	B	------	O	Ile	3	Q	2.56
NZ	Lys	59	B	------	GG1	Ile	3	Q	3.78

Supplementary Figure 9. Interactions between Cas2 and AcrVA5-peptide.

(a) This figure illustrates the specific interactions, including both hydrogen bonds and non-bonded contacts, between the Cas2 and AcrVA5-peptide. In the 2D representation, the Cas2 is depicted in the contact residues (Lys^{55}, Glu^{58} and Lys^{59}), whereas the AcrVA5-peptide is represented using stick models. Hydrogen bond interactions are highlighted with dashed lines. Non-bonded contact points between the peptide and the protein are also illustrated. (b) The Table offers a detailed look at the interaction landscape that governs the complex formation between Cas2 and AcrVA5-peptide.

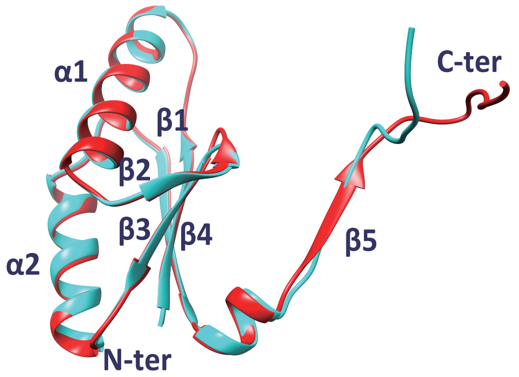

Supplementary Figure 10. Structural superimposition of protomer A and protomer B. Protomer A and protomer B of Cas2 are labeled in red and cyan, respectively.

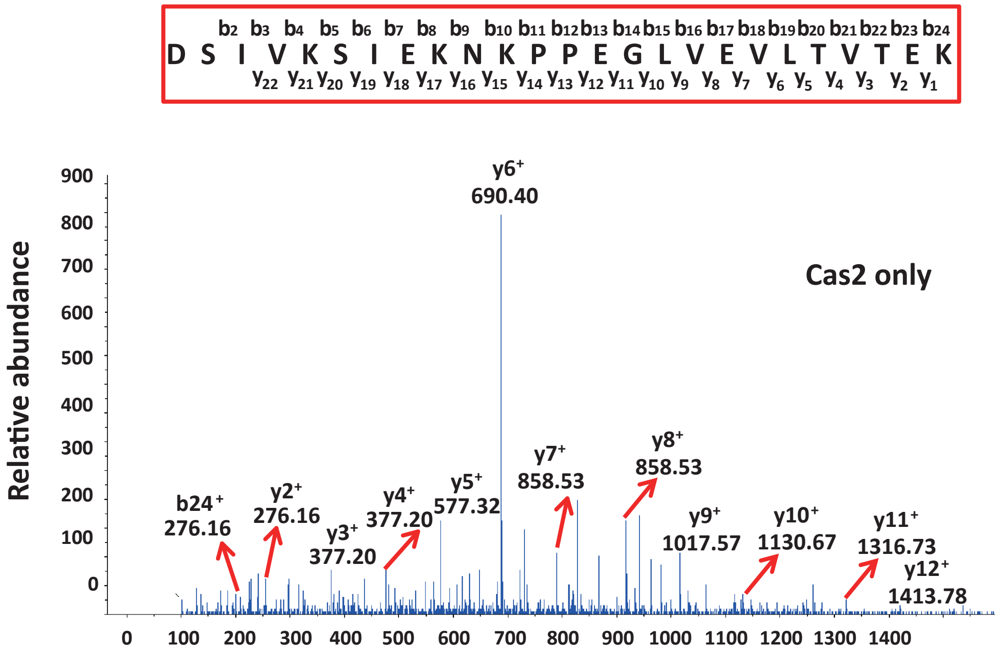

The mass spectrum of Cas2-only expressed in *E. coli*

Supplementary Figure 11. Mass spectrum of Cas2 expression in *E. Coli*.

This figure presents the mass spectrum for Cas2 protein expressed solely in *E. Coli*. The spectrum depicts various peaks. The lack of specific peaks indicative of Lys^{55} modification signifies that no such modification has occurred in the Cas2 protein.

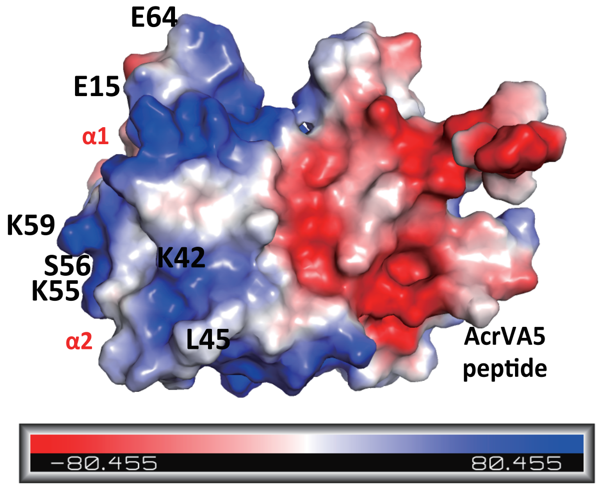

Supplementary Figure 12. Vacuum electrostatics surface view of the complex of Cas2 with AcrVA5 peptide.

The blue and red color represent the positive and negative potential residues, show a continuous positive patch in the surface region as indicated, which show the proximity of the interaction patch to the protospacer binding site. The critical residues, E15, K42, L45, K55, S56, K59 and E64, are labeled.

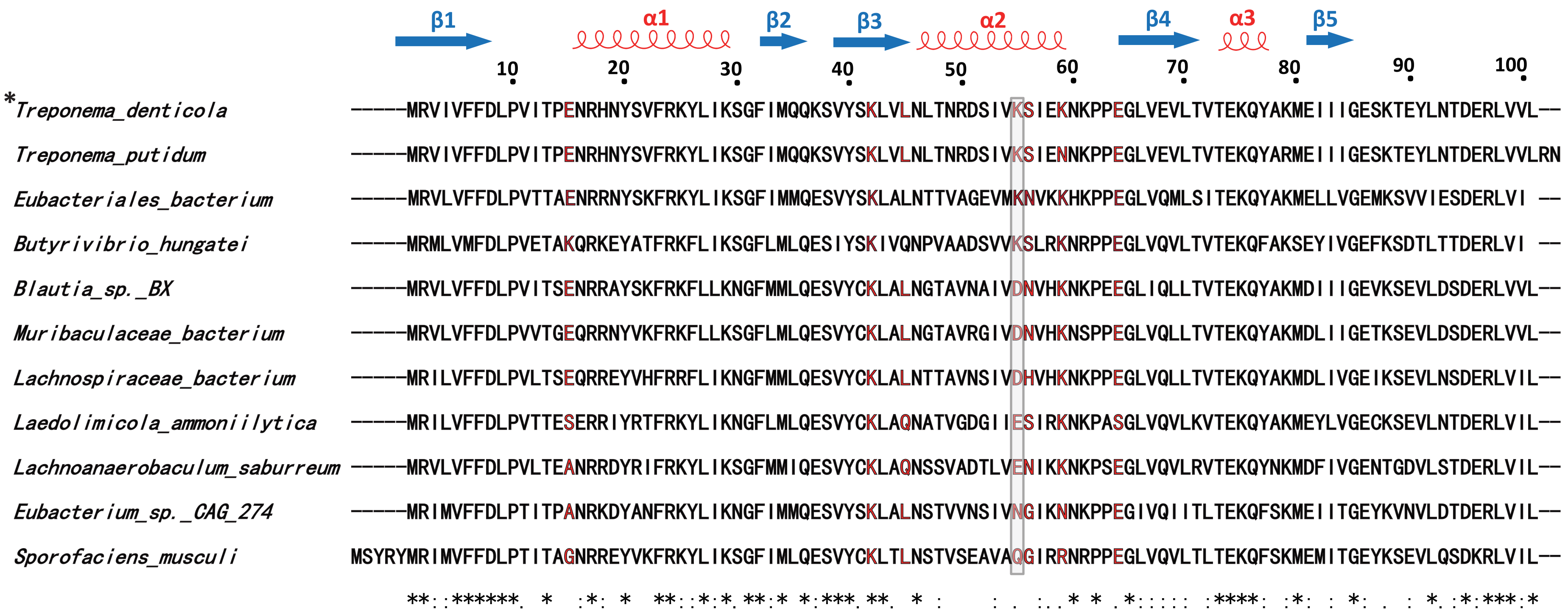

Sequence alignment of Cas2 protein

***Residues numbered according to Cas2 (*Treponema_denticola*) numbering.**

Supplementary Figure 13. Multiple sequence alignment of Cas2 protein.

Using full length Cas2 sequence for the Position-Specific-Iterated (PSI)-BLAST search, divergent species of Cas2 were identified. Sequence alignment was performed using ClustalW. Above the sequence is the secondary structure, α-helices are displayed as helices.

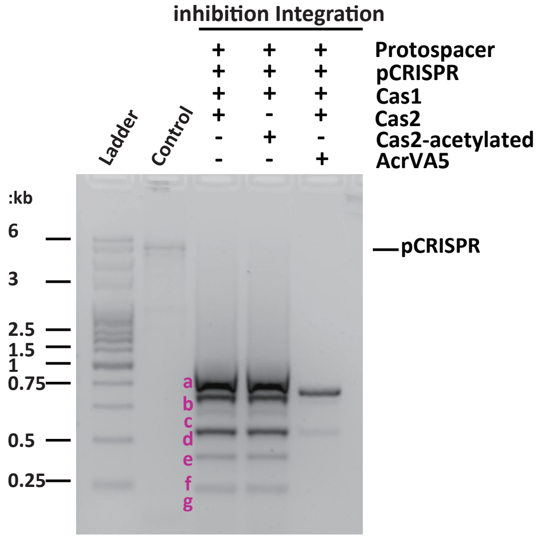

Supplementary Figure 14. ***In Vitro*** **protospacer integration assay and integration inhibition assay.** This figure presents the results of an *in vitro* protospacer integration assay as well as an integration inhibition assay. The assays establish the integration of protospacers under various conditions, specifically focusing on whether the Cas2 protein was acetylated or not. The figure underscores that the acetylation of Lys^{55} did not evidently affect the regulation and control of protospacer integration.
